# Wnt5a Regulates Midbrain Dopaminergic Axon Growth and Guidance

**DOI:** 10.1371/journal.pone.0018373

**Published:** 2011-03-31

**Authors:** Brette D. Blakely, Christopher R. Bye, Chathurini V. Fernando, Malcolm K. Horne, Maria L. Macheda, Steven A. Stacker, Ernest Arenas, Clare L. Parish

**Affiliations:** 1 Florey Neuroscience Institutes, The University of Melbourne, Victoria, Australia; 2 Centre for Neurosciences, The University of Melbourne, Victoria, Australia; 3 St Vincent's Hospital, Fitzroy, Victoria, Australia; 4 Ludwig Institute for Cancer Research, Royal Melbourne Hospital, Parkville, Victoria, Australia; 5 Laboratory of Molecular Neurobiology, Department of Biochemistry and Biophysics, Karolinska Institute, Stockholm, Sweden; Seattle Children's Research Institute, United States of America

## Abstract

During development, precise temporal and spatial gradients are responsible for guiding axons to their appropriate targets. Within the developing ventral midbrain (VM) the cues that guide dopaminergic (DA) axons to their forebrain targets remain to be fully elucidated. Wnts are morphogens that have been identified as axon guidance molecules. Several Wnts are expressed in the VM where they regulate the birth of DA neurons. Here, we describe that a precise temporo-spatial expression of Wnt5a accompanies the development of nigrostriatal projections by VM DA neurons. In mice at E11.5, *Wnt5a* is expressed in the VM where it was found to promote DA neurite and axonal growth in VM primary cultures. By E14.5, when DA axons are approaching their striatal target, Wnt5a causes DA neurite retraction in primary cultures. Co-culture of VM explants with Wnt5a-overexpressing cell aggregates revealed that Wnt5a is capable of repelling DA neurites. Antagonism experiments revealed that the effects of Wnt5a are mediated by the Frizzled receptors and by the small GTPase, Rac1 (a component of the non-canonical Wnt planar cell polarity pathway). Moreover, the effects were specific as they could be blocked by Wnt5a antibody, sFRPs and RYK-Fc. The importance of Wnt5a in DA axon morphogenesis was further verified in *Wnt5a* ^−/−^ mice, where fasciculation of the medial forebrain bundle (MFB) as well as the density of DA neurites in the MFB and striatal terminals were disrupted. Thus, our results identify a novel role of Wnt5a in DA axon growth and guidance.

## Introduction

Dopamine (DA) neurons within the ventral midbrain (VM) project to the striatum and prefrontal cortex forming the nigrostriatal, mesocortical and mesolimbic pathways, which are important for motor and cognitive functions. DA neuron dysfunction is associated with a number of neurological and psychiatric disorders. Abnormal development of the nervous system may contribute to these disorders; hence, the importance of understanding the processes involved in DA neuron maturation and connectivity. Whilst the cues that orchestrate the birth of midbrain DA neurons are well established, the signals regulating DA neurite morphogenesis (including neurite growth, axon guidance and synaptogenesis) are less well defined.

Several studies have identified cellular and molecular signals that participate in establishing these pathways (see review by [1]), including Ephrins [2]–[4], Semaphorins [5]–[9], Netrins and Slits [10], [11], Engrailed-1 [12], [13], and Sonic hedgehog [14]. In this study we asked whether Wnts also regulate DA axon morphogenesis.

Wnt1 and Wnt5a are important morphogens for VM development, regulating proliferation, differentiation and survival of DA neurons [15]–[24]. Wnts also participate in axon guidance elsewhere in the central nervous system [25]–[30]. Specifically, Wnt5a repels corticospinal axons [31]–[33], commissural axons [34] and cortical axons in the corpus callosum [35], [36], and promotes neurite elongation of cortical neurons [33].

Wnt5a is a highly conserved diffusible protein whose signal is transduced by Frizzled (Fz) receptors and/or co-receptors including the atypical tyrosine kinases Ryk and Ror2. Dependent on the receptor and cell type, Wnt5a has been shown to activate three signaling pathways: the Wnt/β-catenin/canonical pathway, the Wnt/calcium/non-canonical pathway, and the Wnt/planar cell polarity (PCP)/non-canonical pathway [37]–[39]. However, little is known about which of these pathways and downstream signaling components mediate Wnt5a's influence on axon growth and guidance. Moreover, it is not known whether Wnt5a promotes neuritogenesis and axonal growth of DA axons in the nigrostriatal system. We therefore set out to determine whether Wnt5a plays a role in DA axon growth and guidance and examined the involvement of some of the candidate Wnt5a receptors and Wnt signaling components.

## Materials and Methods

### Animals

#### Ethics statement

This study conformed to the Australian National Health and Medical Research Council's published Code of Practice for the Use of Animals in Research, and experiments were approved by the Florey Neuroscience Institutes animal ethics committee (#07-040).

Embryos were isolated from time-mated C57BL/6 mice or Sprague Dawley rats. Animals were time mated overnight and visualization of a vaginal plug on the following morning was taken as embryonic day (E) 0.5. B6;129S7-*Wnt5a^tm1Amc^*/J (subsequently referred to as *Wnt5a* ^−/−^ mice) were obtained from Jackson Laboratories (JAX, ® Strain 004758) and maintained on a mixed B6:129 background [40]. Wnt5a embryos were collected at E12 and E18.

### In situ hybridization and immunohistochemistry

Embryos were isolated in ice-cold PBS, fixed overnight in 4% paraformaldehyde, followed by overnight immersion in 30% sucrose in PBS. Embryonic day 11.5 (E11.5), E12 and E14.5 embryos were cryosectioned on either a sagittal or coronal plane at a thickness of 14 µm. E18 embryos were cryosectioned at 16 µm. In situ hybridization (ISH) was performed as previously described [41], using a DIG-labelled single-stranded RNA probe for Wnt5a [40]. Following ISH, the tissue was again fixed using 4% paraformaldehyde prior to immunohistochemistry for tyrosine hydroxylase (TH; the rate-limiting enzyme in dopamine synthesis and marker of DA neurons and neurites).

Immunohistochemistry was performed on 4% paraformaldehyde-fixed cultures and slides as previously described [20]. The following primary antibodies were used: rabbit anti-TH (1∶250 or 1∶1500, PelFreez); sheep anti-TH (1∶500, PelFreez); mouse anti-βIII-tubulin (TUJ1; 1∶1000, Promega). Appropriate fluorophore-conjugated (Cy2 and Cy3, Jackson ImmunoResearch Laboratories) secondary antibodies (or biotinylated secondary antibody together with the Vector Laboratories ABC immunoperoxidase kit) were used for visualization.

### Quantitative real-time PCR

Given the lack of reliable antibodies to detect many of the Wnt ligands and receptors histochemically, we relied on quantitative real-time PCR (Q-PCR) to assess the expression of Wnt5a, Ryk and Fz3 within VM, and more specifically within DA neurons. Ventral midbrains were isolated and dissociated from E11.5 Tyrosine Hydroxylase-GFP (TH-GFP) reporter mice, in which all DA neurons express GFP [42]. Dissections are described in further detail below. At least five TH-GFP^+^ embryos were used for each dissection with four independent dissections performed. Using previously described methods [43], fluorescence-activated cell sorting (FACS) was used to separate GFP^+^ cells (dopamine neurons) from GFP^−^ cells (non-TH^+^ neurons within the VM) in order to identify the source of Wnt5a, Ryk and Frizzled-3 in the midbrain. Following sorting, total RNA was isolated using the PicoPure kit (Arcturus). Alternatively, the ventral midbrain (VM), dorsal midbrain (DM) and the rest of the embryo (E) were microdissected from four independent E11.5 mouse litters. Following tissue isolation, total RNA was isolated using the RNeasy Micro kit (Qiagen).

RNA was reverse transcribed using Superscript III First-Strand Synthesis supermix for qRT-PCR (Invitrogen) and Q-PCR was carried out using the SYBR GreenER^TM^ qPCR SuperMix Universal (Invitrogen) on an ABI7700 sequence detection system (Applied Biosystems, Foster City, CA) using the comparative ΔΔCT method [44]. Oligonucleotide sequences were as follows:

*HPRT* forward, 5′- CTTTGCTGACCTGCTGGATT -3′

*HPRT* reverse, 5′- TATGTCCCCCGTTGACTGAT -3 ′

*Wnt5a* forward, 5′- AATAACCCTGTTCAGATGTCA -3′

*Wnt5a* reverse, 5′- TACTGCATGTGGTCCTGATA -3′

*Ryk* forward, 5′- CGCTCTGTCCTTTAACCTGC -3′

*Ryk* reverse, 5′- CCAGTTCAATCCTTTTCATGC -3′

*Fz3* forward, 5′- CAGTCTGCTACATGAGGTG -3′

*Fz3* reverse, 5′- CGCCACTAATATTGTCACCT -3′

### Ventral Midbrain Primary Cultures

The ventral midbrain of E11.5 and E14.5 mouse (or E13.5 rat) embryos was microdissected in chilled L15 media (Invitrogen). Note, stages in development of the dopamine systems occur approximately 2 days later in rats than mice, hence E13.5 rat is considered equivalent to E11.5 mouse. Whilst initial studies were performed in mice, they were verified later in rats. Rat embryos were used in all antagonism studies as greater volumes of VM primary neurons can be obtained, necessary for the outlined antagonism studies that required multiple conditions. The isolated ventral midbrains were enzymatically dissociated in HBSS containing 0.05% trypsin and 0.1% DNase for 12 minutes at 37°C. Cells were subsequently centrifuged and resuspended in serum-free N2 medium consisting of a 1∶1 mixture of F12 and MEM supplemented with 15 mM HEPES buffer, 1 mM glutamine, 6 mg/ml glucose (Sigma-Aldrich), 1 mg/ml bovine serum albumin and N2 supplement (all purchased from Invitrogen). Cells were seeded at a density of 125,000 cells per well in a 48-well plate at 37°C, 5% CO_2_ for 72 hours.

Wnt5a recombinant protein (R&D Systems) was added to the cultures at the time of cell seeding. For antagonism experiments using secreted frizzled-related protein 1 (sFRP-1; 5 µg/ml, R&D Systems), Wnt5a antibody (αWnt5a; 2 µg/ml, R&D Systems), human RYK-Fc (3 µg/ml, see details below), goat anti-Frizzled3-CRD (αFz3-CRD; 3 µg/ml, R&D Systems), Dickkopf-1 (Dkk1; 500 ng/ml, R&D Systems), casein kinase 1 inhibitor (D4476, 50 mM, Roche) or Rac-1 inhibitor (NSC233766, 500 nM, Calbiochem), Wnt5a and the antagonist were added to the wells 15 minutes prior to seeding the VM cells.

To generate the RYK-Fc, the human RYK WIF domain (residues 60–195 of Genbank accession number NP_002949.2) was subcloned by PCR into pApex-3.Fc.FLAG, between an IL-3 signal peptide and the human IgG_1_ Fc domain, to create a fusion protein with a carboxyl-terminal FLAG epitope tag. CHO-K1 cells were transfected with pApex-3.hRYKWD.Fc.FLAG using FuGENE 6 (Invitrogen) and selection applied after 24 h (200 µg/ml hygromycin B; Invitrogen). Stable colonies were picked after 7–9 days. The stable cell line hRYKWD.Fc.FLAG/CHO was seeded into a medium FiberCell cartridge, 20 kDa (FiberCell Systems), using DMEM (Invitrogen), 10% fetal bovine serum (FBS) and 100 µg/ml hygromycin B. Extracapillary space media from the FiberCell cartridge was collected every 2–3 d, filtered using 0.22 µm filters (Millipore), and secreted protein was purified using anti-FLAG M2 affinity gel (Sigma) as previously described [45].

TH-immunoreactive (TH^+^) neurons from each primary VM culture were analyzed from 3–5 independent cultures. Under all culture conditions, sampling was commenced in the second field of view from the left-hand side of the culture well. The first 30 TH^+^ cells found to be measurable (neurites intact and distinguishable from other stained neurites, i.e. not intertwined with other TH^+^ neurites) were quantified in order to avoid any potential sampling bias. In each experiment, data was compared to the mean normalized control value (set at 100%) to account for inter-experimental variation. Photomicrographs of each DA neuron (identified by TH^+^) were taken using a 20× objective (Olympus IX71) and the following measurements obtained using NeuronJ software (ImageJ, NeuronJ plugin, NIH): the total numbers of neurites per DA neuron, the number of neurite branches, total length of all neurites per neuron and the length of the dominant neurite (the longest, most dominant neurite arising from the soma, and thereby presumably the axon [46]).

VM primary cultures were also performed from *Wnt5a* ^−/−^ and littermate *Wnt5a* ^+/+^ and *Wnt5a* ^+/−^ mice. Given that single VM were required for each culture, and the low yield of neurons generated, each dissected VM was dissociated as describe above and plated into a 96-well plate (50,000 cells/well). Cells were cultured for three divisions (3DIV) prior to staining and measurements of TH^+^ neurons. *Wnt5a* ^−/−^ cultures were compared to *Wnt5a* ^+/+^ and *Wnt5a* ^+/−^ littermates.

### Immunoblotting

SN4741 cells were cultured in DMEM, 10% FBS, L-glutamine (2 mM), penicillin/streptomycin (50 U/ml) and glucose (0.6%). For analysis of intracellular Wnt signaling, 100,000 cells were seeded in 12-well plates, grown overnight in the absence of serum and stimulated for 2 hours in the same media with Wnt5a (0, 30, 100, 300, 1000 ng/ml; R&D Systems), Wnt5a (300 ng/ml) + RYK-Fc (3 µg/ml) or Wnt5a (300 ng/ml) + αFz3-CRD (3 µg/ml; R&D Systems). Preparation of lysates and immunoblotting were carried out as previously described [47]. The following primary antibodies were used: rabbit anti-Dvl2 (1∶500, Santa Cruz) and mouse anti-β-actin (1∶3000, Sigma).

### Co-culture explant assays

Neuronal c17.2 cells over-expressing Wnt5a (or the parental cell line containing the empty vector i.e. mock) were cultured as previously described [48]. Aggregates of the c17.2 cells were generated by plating 50,000 cells per 20 µl droplet onto the inverted lid of a 60 mm culture plate containing 1 ml PBS (to maintain humidity). Cell aggregates formed within 48 hours and were floated in N2 media prior to co-culture with VM explants. The ventral midbrain of E11.5 or E14.5 mice was isolated in L15 media. Each VM was cut into approximately four segments.

To prepare a stock of collagen gel matrix for culture experiments, 10 mg of rat tail collagen (Roche) was dissolved in 3 ml of 0.2% acetic acid. To polymerize the collagen, the collagen solution was mixed with 0.2 M HEPES at a ratio of 8∶1∶1 and finally pH adjusted using 1 M NaOH. VM explants were plated onto the culture dishes (24-well plate) and excess media removed. 50 µl of the collagen gel was then applied to the explant. Aggregates of c17.2 cells over-expressing Wnt5a (or mock-transfected cells) were inserted in the gel matrices approximately 300–500 µm from the explants. The collagen was allowed to polymerize at 37°C for 20 minutes. After polymerization of the gel, 500 µl N2 media was added to each well and left in culture for 72 hours. Explants were fixed in 4% paraformaldehyde for 30 minutes prior to immunocytochemistry. To quantify chemoattraction, the field was divided into four orthogonal quadrants and the number of TH^+^ fibers in the distal (D) and proximal (P) quadrants, with respect to the cell aggregate, were counted. For each explant, the proximal:distal ratio was calculated and used as a chemotaxic index. For antagonism of the chemotaxic effects of Wnt5a, casein kinase 1 inhibitor (D4476; 50 mM), Rac1 inhibitor, (NSC23766; 500 nM) or anti-Fz3-CRD (3 ug/ml) were added to the cultures at the same time as N2 media.

### Analysis of *Wnt5a* ^−/−^ mice

All *Wnt5a* ^−/−^ embryos were compared to littermate controls, wildtype *Wnt5a* ^+/+^ and heterozygotes *Wnt5a* ^+/−^, n = 4–7 embryos per genotype. Given the postnatally lethal phenotype of the *Wnt5a* ^−/−^ mouse, the midbrain dopamine pathways were examined developmentally and quantification performed at E18, an age when the DA pathway is established, axons have reached the striatum and numerous synaptic contacts made. Anatomical changes were also observed within Wnt5a(−/−) and Wnt5a(+/+) littermates at E12.

Changes within the midbrain dopamine pathways in Wnt5a(+/+) and Wnt5a(−/−) mice were observed by chromogenic staining for the TH+ neuron and quantified using Stereoinvestigator software (MicrobrightField, USA) on a Leica DML microscope. In adjacent series, z-stack images of TH+ immunofluorescence were taken on a confocal microscope (Zeiss Pascal) to represent the quantified changes.

At E18, the volume of the medial forebrain bundle (MFB) was estimated by delineating the area of the TH^+^ fiber bundle in the first section rostral to the midbrain TH^+^ neurons until the final section prior to the arrival of TH^+^ fibers in the striatum. The MFB was delineated in approximately 7 sections (16 µm thickness, 1∶10 series, i.e. approximately 1120 µm in length) from each brain, with the area and total volume estimated using StereoInvestigator software.

The number of dopaminergic fibers (TH^+^) in the MFB and the density of TH^+^ varicosities in the lateral striatum were estimated using previously described fractionator methods [49]–[51]. The density of TH^+^ fibers was assessed at two independent levels along the MFB, (i) 320 µm and (ii) 800 µm rostral to the midbrain DA neurons. TH^+^ fiber counts were made at regular pre-determined intervals (x = 50 µm, y = 50 µm). These counts were derived by means of a grid program, through which a systematic sample of the area occupied by the fibers was made from a random starting point. An unbiased counting frame of known area (7 µm×7 µm = 49 µm^2^) was superimposed on the image of the tissue sections viewed under a 100×, N.A. 1.30 oil immersion objective. The number of TH^+^ fibers at each level was counted in 16 µm thick, 1∶10 serial sections.

TH^+^ varicosities in the lateral 400 µm of the striatum were counted from 16 µm serial sections, 1∶10 series, with four sections sampled from each striatum. Counts of TH^+^ varicosities were made at regular predetermined intervals (x = 150 µm, y = 150 µm) using an unbiased counting frame of known area (6 µm×6 µm = 36 µm^2^). TH^+^ varicosities were identified as predominantly round swellings in association with axonal processes. TH^+^ varicosity counts were expressed as terminal density, with comparisons made between *Wnt5a* ^+/+^, *Wnt5a* ^+/−^ and *Wnt5a* ^−/−^ mice. For TH^+^ fiber counts and TH^+^ varicosity numbers, the coefficients of error (CE) and coefficients of variance (CV) were calculated as estimates of precision, and values of less than 0.1 were accepted [50], [52],[53].

### Statistical analysis

One-way ANOVAs with Tukey post-hoc tests or Student's t-tests were used to identify statistically significant changes. Statistical significance was set at a level of p<0.05. Data represents mean ± s.e.m.

## Results

### Wnt5a is expressed along the developing nigrostriatal pathway

**Figure 1:**
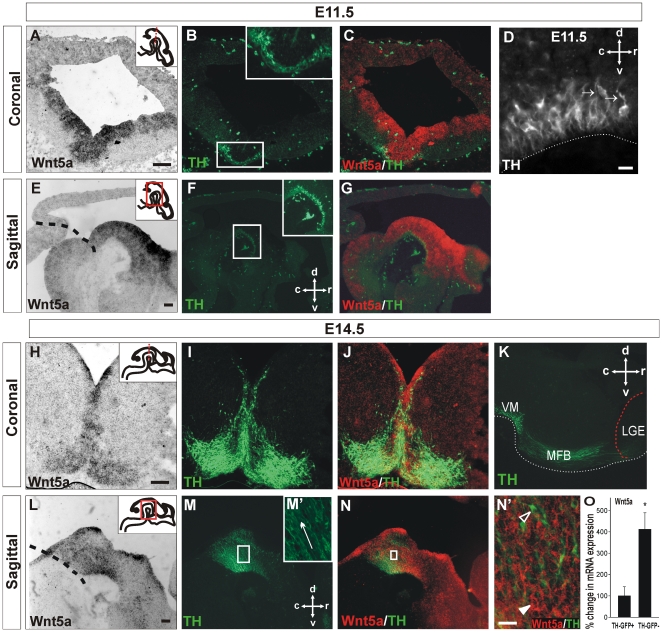
Wnt5a expression in the ontogeny of the midbrain DA axon. (**A**) In situ hybridization in a coronal section of the mouse midbrain showed Wnt5a expression overlapping with (**B**) TH^+^ cells in the developing ventral midbrain at E11.5, during the period of initiation of neurite outgrowth. (**C**) Merged image of TH and Wnt5 expression. (**D**) Sagittal section of mouse VM at E11.5 illustrating TH^+^ fibers polarized towards the DM, as indicated by the arrows. (**E**) Sagittal section at E11.5 revealed a high rostral to low caudal gradient of Wnt5a in the VM, (**F**) surrounding the DA neurons and neurites as well as higher expression at the ventricular zone compared to the mantle zone. (**G**) Merged image of TH and Wnt5 expression depicted in E and F. (**H**) At E14.5, during maturation of the midbrain dopamine pathways, a coronal section revealed Wnt5a expression was maintained in the VM, overlapping with (**I**) the TH^+^ cells. (**J**) Merged image of TH and Wnt5 expression. (**K**) Photomicrograph illustrating DA fibers in the MFB approaching the LGE at E14.5. (**L**–**N**) Sagittal section (medial to panel (**K**)) illustrating reversal of the Wnt5a gradient, with Wnt5a expression greater in the caudal VM than rostral VM. (**M**') Enlargement from (**M**) illustrating dorsal trajectory of TH^+^ fibers (**N**') Enlargement from (**N**) illustrating that Wnt5a is most likely secreted from TH^−^ cells. Filled arrow-head: Wnt5a-labeled cell (red). Unfilled arrow-head: TH^+^ neuron (green). (**O**) Dissected VM from TH-GFP mice were analyzed by flow cytometry and sorted into GFP^+^ (TH^+^ neurons) and GFP^−^ (non-DA neurons). Q-PCR revealed that the majority of Wnt5a expression was not in the DA neurons (TH-GFP^-^ fraction). Scale bars: A–C,E–G,H–J,L–N = 100 µm; D,N' = 25 µm. Data represents mean ± s.e.m., n = 5, * p<0.05. Black dashed line (panels E,L) represents the midbrain-hindbrain boundary.

The first ventral midbrain DA neurons are born in mice at E10.5 and shortly thereafter, at E11.5, the first DA neurites appear. Initially, these neurites project dorsally towards the dorsal midbrain (Fig. 1D) and are subsequently deflected rostrally towards their forebrain targets [54]. The first DA neurites approach the border of the ventrolateral ganglionic eminence at E14.5 (Fig. 1K) and increase in number without further elongation (axonal stalling). Subsequently these fibers enter the ventral areas of the lateral ganglionic eminence (LGE; the future striatum), followed by lateral, and finally medial and dorsal regions of the LGE [1]. A smaller subset of DA axons arising from the VM project to the prefrontal cortex. Upon arrival, DA fibers make synaptic contacts throughout the LGE and cortex, with axonal branching, pruning and synaptogenesis continuing into the first weeks of postnatal development.

In order to assess the possible role of Wnt5a in DA neuritogenesis and axon formation, we first examined the temporal and spatial expression of Wnt5a relative to the developing DA pathway in the MFB; expanding on previous studies by Andersson et al., 2008 who examined Wnt5a expression in relation to the birth of DA neurons [23]. Using in situ hybridization to detect Wnt5a expression and immunohistochemistry against TH (to identify DA neurons), strong Wnt5a expression was apparent within the VM where DA neurons reside at both E11.5 and E14.5 (Fig. 1A–C,E–G,H–J,L–N). At E11.5, Wnt5a expression was greater in the ventricular zone (Fig. 1E), whilst at E14.5 expression was greater in the mantle zone of the VM (Fig. 1L). Closer examination of the E11.5 VM revealed a rostro-caudal gradient of Wnt5a, with levels higher in the rostral VM than caudal VM (Fig. 1E). By E14.5 this gradient was reversed, with rostral VM expression decreased and Wnt5a expression higher in the caudal midbrain (Fig. 1L compared to 1E). At this stage, neurites maintained their initial dorsal projection (Fig. 1M, M'), but subsequently projected rostrally (Fig. 1K), away from the strong ventral source of Wnt5a in the VM.

Wnt5a mRNA expression was more closely examined in VM cells isolated by FACS from the TH-GFP reporter mouse [42]. Quantitative real-time PCR (Q-PCR) performed on the GFP^+^ fraction (DA neurons) and GFP^−^ fraction (other VM cells) revealed that Wnt5a mRNA expression was significantly higher in the GFP^−^ fraction (four-fold increase, p = 0.042) compared to the GFP^+^ fraction (Fig. 1O). These findings were in accordance with Wnt5a in situ hybridization, with expression greatest in non-TH^+^ cells (Fig. 1N', filled arrow-head). These results are also in agreement with previous studies showing greater expression of Wnt5a in glial cells (radial glia first and later astrocytes) compared to neurons in the developing VM [22], [56].

### Wnt5a increases dominant DA neurite length and reduces DA neurite branching in VM cultures at E11.5, but not at E14.5

As the temporal-spatial pattern of expression of Wnt5a was appropriate for a role in DA neurite development, we tested the effect of Wnt5a on DA neurite growth by applying recombinant Wnt5a protein to VM primary neuron cultures isolated from E11.5 and E14.5 mouse embryos and examining the neurites of tyrosine hydroxylase (TH; rate-limiting enzyme in DA synthesis and marker of DA neurons) immunoreactive neurons.

**Figure 2:**
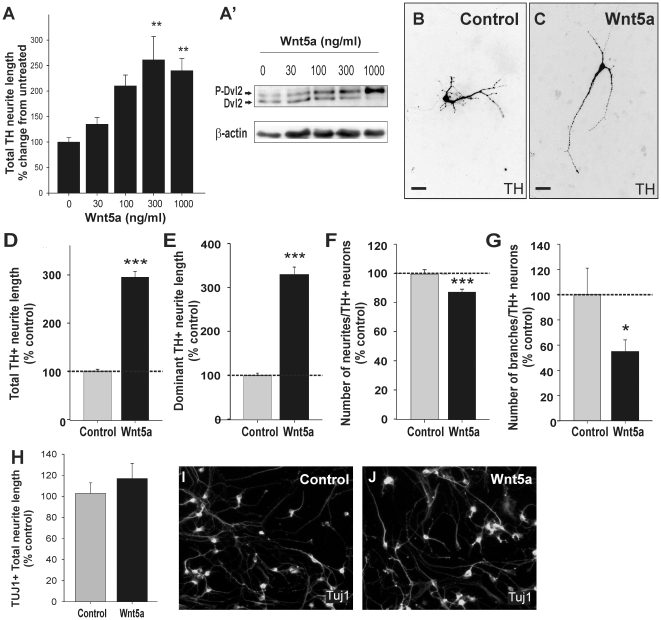
Wnt5a promotes DA axon elongation and alters neuron complexity during the period of initiation of neurite outgrowth. (**A**) Wnt5a recombinant protein promoted TH^+^ neurite elongation in a dose-responsive manner in mouse E11.5 VM primary cultures. (**A**') Wnt5a activated Dvl2 in a dose-responsive manner in the SN4741 dopaminergic cell line. Note the mobility shift of the Dvl2 protein with increasing doses of Wnt5a. (**B**) Photomicrographs illustrating the complexity of DA neurons under control conditions, and (**C**) following Wnt5a treatment. (**D**) Wnt5a induced a three-fold increase in total neurite length compared to control. (**E**) The effect of Wnt5a was specific to the dominant neurite (presumably the DA axon). Wnt5a protein reduced the number of (**F**) DA neurites and (**G**) DA neuritic branches per neuron. (**H**) Immunocytochemistry for TUJ1 revealed that the effects of Wnt5a within the VM were specific to DA neurons, with no change in neurite length observed for other neurons in culture. (**I**) Compared to control cultures, (**J**) Wnt5a had no effect on neurite length of TUJ-labeled cells. Cells were analyzed after 3DIV. Scale bar = 25 µm. Data represents mean ± s.e.m., n = 4–5 cultures; * p<0.05, ** p<0.01, *** p<0.001.

A dose-response curve revealed that Wnt5a promotes DA neurite elongation in a dose-dependent manner in E11.5 VM cultures. Maximal elongation, as measured by total neurite length, was achieved with a dose of 300 ng/ml of Wnt5a (Fig. 2A). Immunoblots were performed in a dopaminergic cell line (SN4741) in order to confirm that Wnt5a induced intracellular activation of Wnt signaling. We found that 100, 300 and 1000 ng/ml of Wnt5a induced dishevelled-2 (Dvl2) phosphorylation (visible by Western blot as a mobility shift of the protein) in a dose-dependent manner (Fig. 2A'). The effects were optimal at 300 ng/ml and this dose was used for further assessment of the neurite arbors of TH^+^ cells. Wnt5a treatment of E11.5 VM cultures increased total neurite length compared to control treated cultures (295%±12%, p<0.001; Fig. 2B–D). Other morphological changes were also observed in Wnt5a treated cultures. The dominant neurite was significantly longer compared to controls (330%±17%, p<0.001; Fig. 2E). Furthermore, Wnt5a treatment resulted in fewer neurites (88%±2%, p<0.001; Fig. 2B,C,F) and branches (55%±9%, p = 0.012; Fig. 2B,C,G) compared to controls, suggesting that Wnt5a promotes the extension of DA axons, rather than the elaboration of shorter neurites or dendritic trees [46]. These findings were also replicated in E13.5 rat cultures (comparable in age to mouse E11.5), demonstrating conservation of the Wnt5a effect across species (data not shown).

We next examined the specificity of the effects of Wnt5a by examining the neurite length of βIII-tubulin immunoreactive (TUJ1^+^) neurons within the culture, knowing that TH^+^ cells represent approximately only 5% of the neurons in the VM culture. The total length of TUJ1-labeled neurites in cultures treated with Wnt5a were not significantly longer than neurites in control cultures (117%±14%, and 100%±10%, respectively, p = 0.309; Fig. 2H–J), confirming that Wnt5a selectively affected DA neurites.

**Figure 3:**
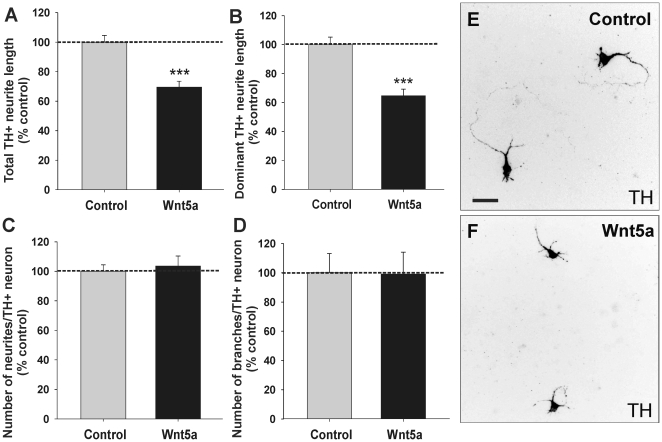
Wnt5a causes DA neurite retraction in older ventral midbrain cultures. (**A**) At a time when DA axons would normally be approaching their striatal targets (mouse E14.5), treatment with Wnt5a protein caused retraction of TH^+^ neurites, and more specifically (**B**) DA axons (dominant neurite length). Wnt5a had no effect on the complexity of DA neurons, as assessed by (**C**) neurite number and (**D**) neurite branching. Photomicrographs illustrating examples of neurite retraction following Wnt5a application (**F**), compared to control (**E**). Cells were analyzed after 3DIV. Scale bar = 25 µm. Data represents mean ± s.e.m., n = 4–5 cultures, *** p<0.001.

Surprisingly, when the activity of Wnt5a (300 ng/mL) was examined on older (E14.5) VM primary cultures, the effects on DA neurite length were reversed. Total neurite length was significantly reduced (69%±4.0%, Fig. 3A, E–F) and the length of the dominant process (axon) was also decreased compared to control-treated cultures (65%±5%; Fig. 3B). Furthermore, Wnt5a treatment affected neither the number of DA neurites nor their branching (Fig. 3C,D). Collectively, these results indicate that Wnt5a differentially regulates DA neurite growth and morphology during development.

### The effects of Wnt5a protein on DA neuritogenesis are specific and mediated by Frizzled

**Figure 4:**
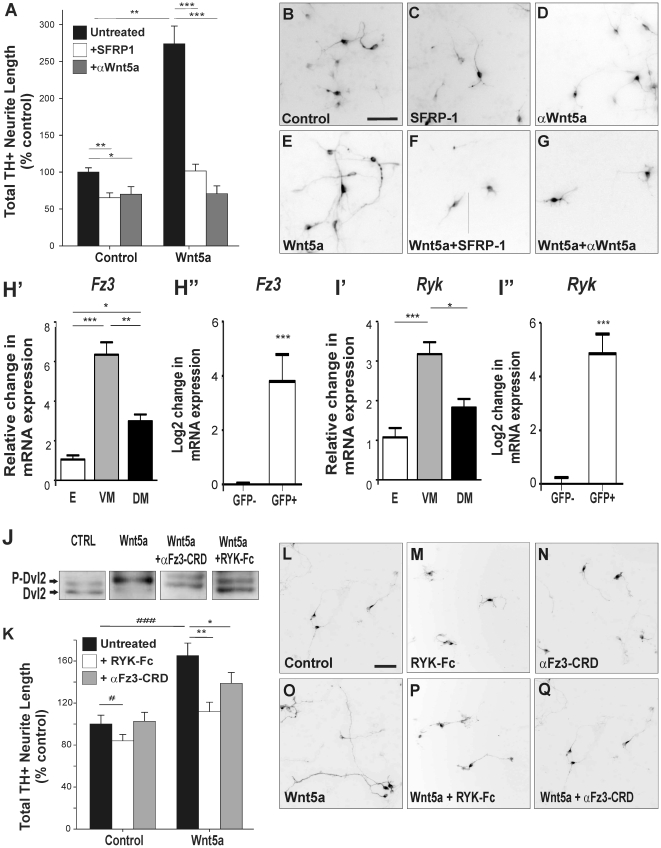
The effects of Wnt5a protein on DA neuritogenesis are specific and mediated by Frizzled. (**A**) Whilst sFRP-1 and Wnt5a blocking antibody (αWnt5a) were capable of reducing neurite length in E13.5 VM rat primary cultures under control conditions (presumably due to antagonism of endogenous Wnt signaling), in the presence of Wnt5a they significantly reduced neurite length compared to Wnt5a alone. (**B**–**G**) Examples of Wnt antagonism in VM cultures ± Wnt5a treatment. Q-PCR analysis revealed that the Wnt-related receptors Fz3 (**H**') and Ryk (**I**') were highly expressed in the VM compared to the dorsal midbrain (DM) and whole embryo (E). Furthermore, Fz3 (**H”**) and Ryk (**I”**) showed significantly higher expression within DA neurons (GFP^+^) compared to other cells (GFP^−^) isolated from the VM of TH-GFP mice. (**J**) Wnt5a (300 ng/ml) activated Dvl2 in SN4741 cells, an effect that could be blocked by αFz3-CRD (3 µg/ml) or RYK-Fc (3 µg/ml). (**K**) E13.5 rat primary VM cultures showed that the effects of Wnt5a on DA neurite length were specific, illustrated by antagonism with RYK-Fc, and mediated through the Fz3 receptors, illustrated by blocking of Fz3 with αFz3-CRD. (**L-Q**) Photomicrographs illustrating the effects of Wnt5a ± αFz3-CRD or RYK-Fc on DA neurite length. Cells were cultured for 3DIV. Scale bar  = 100 µm. Data represents mean ± s.e.m., n = 4–5 cultures. Significantly different from control: #p<0.05, ### p<0.001. Significantly different from Wnt5a: * p<0.05, ** p<0.005.

To confirm the specificity of the effects of Wnt5a on DA neurite development, we treated primary VM cultures with different Wnt blocking tools and subsequently evaluated TH^+^ neurites. Given the maintained effect of Wnt5a on DA neurites in both mice and rats, we performed these antagonism experiments in rats due to the increased yield of VM tissue, and the numerous antagonists to be employed. Figure S1 provides a schematic representation of the site of action of these various antagonists. Secreted Frizzled-related proteins (sFRPs) modulate Wnt signaling by preventing Wnt from interacting with membrane-bound receptors. In the absence of exogenous Wnt5a, sFRP-1 reduced neurite length to 62%±4% compared to untreated cultures (Fig. 4A–C), presumably through antagonism of endogenous Wnt signaling within the VM. This was confirmed by using a Wnt5a blocking antibody (αWnt5a), which also reduced neurite length to 67%±8% (Fig. 4A–B,D). In the presence of Wnt5a, increased neurite length of TH^+^ cells (267%±31%) was completely blocked by co-administration of sFRP-1 (101%±10%) or the αWnt5a (70%±11%; Fig. 4A,E–G). Interestingly, sFRP-2, but not sFRP-3 (data not shown), also antagonized the effects of Wnt5a on neuritogenesis. These results indicated that the effects of exogenously supplied Wnt5a on TH^+^ cells are specific and suggest a role for Wnt5a in DA neuritogenesis.

Previous studies have shown that Wnt5a modulates axon growth and guidance in other systems through interactions with Frizzled receptors and the atypical tyrosine kinase receptor, Ryk [31], [33], [35]. Moreover, Fz3 has been found to be relevant to the development of the DA nigrostriatal pathway as Fz3 expression increases at the time of DA axon extension in the VM [43] and the nigrostriatal pathway was absent in *Fz3* ^−/−^ mice [57], [58]. We thus first examined the expression of Fz3 and Ryk in the VM by Q-PCR and found elevated expression of both receptors in the VM compared to the dorsal midbrain (DM) and the rest of the embryo (E) (Fig. 4H',I'). Further, Q-PCR performed on the GFP^+^ and GFP^−^ fraction of VM tissue isolated from TH-GFP mice, revealed that these receptors were expressed on the DA neurons (GFP^+^) and not surrounding cells (GFP^−^) within the VM (Fig. 4H” and 4I”).

Subsequently, we examined whether E13.5 rat VM cultures treated with antibodies against Fz3 (αFz3-CRD) or in the presence of a Ryk construct containing the human RYK WIF domain (RYK-Fc), blocked the effects of Wnt5a (Fig. 4K–Q). Interestingly, the increase in neurite length produced by the addition of Wnt5a (166%±10%) was significantly attenuated in the presence of a RYK-Fc, to levels not significantly different to control (97%±4%), yet they had no effect on neurite number (data not shown). Similarly, but more modestly, αFz3-CRD also reduced the effect of Wnt5a on total neurite length (from 166%±10% to 129%±6%), but not neurite number (data not shown). Since RYK-Fc can bind Wnt, we interpret that the blocking by RYK-Fc likely mediated by Wnt5a binding. However, as the RYK-Fc construct is not capable of interacting directly at the receptor membrane level, this data should be interpreted with caution. In fact, a recent study has shown no changes in the morphology or trajectory of DA axons in Ryk(−/−) embryos [55]. We believe that additional experiments, employing selective functional blocking antibodies against the Ryk receptor, as well as Ryk-Wnt5a double knock out mice will be required to ascertain whether Ryk plays a role in Wnt5a mediated DA axon growth and guidance.

In contrast, since the αFz3-CRD binds directly to the receptor (with its specificity and function verified by the manufacturer and others Endo 2008), our results indicate that the action of Wnt5a on DA neurite morphology is mediated, at least in part, by the Frizzled-3 receptor. Interestingly, we also found that these proteins antagonized the Wnt5a-mediated Dvl-2 phosphorylation in a dopaminergic cell line (SN4741 cells, Fig. 4J), reinforcing the idea that the effects of Wnt5a require Wnt5a binding and are mediated by Frizzled. It is also important to note that RYK-Fc and αFz3-CRD had no effect on non-DA neurites within the culture, illustrating the specificity of the effects of Wnt5a for DA neurites and the lack of toxicity of the proteins used here (Figure S2).

### Wnt5a regulates neurite morphogenesis in midbrain DA neurons via Rac1

**Figure 5:**
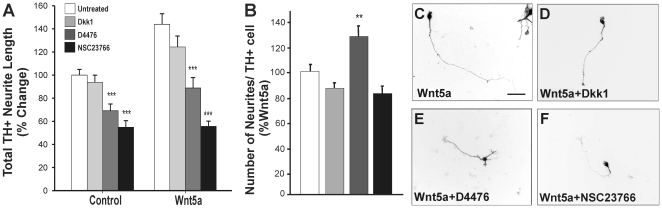
The effects of Wnt5a on elongation are mediated through Rac1 of the non-canonical Wnt/PCP pathway. Select antagonists were used to identify the downstream signaling pathway responsible for mediating the effects of Wnt5a on DA neurites. Antagonism of the canonical Wnt pathway (using Dkk1 recombinant protein), the canonical and non-canonical Wnt pathways (using casein kinase 1 inhibitor, D4476) and even more selectively the non-canonical PCP pathway (using Rac1 inhibitor, NSC23766) revealed that the effect of Wnt5a on (**A**) neurite length and (**B**) neurite number were mediated by the non-canonical PCP pathway (D4476 and NSC23766 both significantly antagonizing the effects of Wnt5a). (**C**–**F**) Examples of changes in DA neuron morphology following treatment with Wnt5a ± selective antagonists. Scale bar  = 50 µm. Data represents mean ± s.e.m., n = 4–5 cultures; ** p<0.01, *** p<0.001.

Depending on the cell type and context, Wnt5a activates either Wnt/β-catenin or Wnt/PCP signaling. However, we previously reported that in dopaminergic cell lines and expanded VM cultures [19], [20] Wnt5a does not activate Wnt/β-catenin signaling. Moreover, Wnt5a mediates axon guidance through non-canonical Wnt pathways in other neuronal systems [33], [59]. To characterize the pathway that mediates the effects of Wnt5a on DA neuritogenesis, we employed an antagonist of the canonical pathway, Dickkopf-1 (Dkk1), to prevent Wnt interaction with the Fz/LRP receptor complex [60], and a casein kinase 1 antagonist, D4476, which blocks both the canonical and non-canonical pathways [61]. While Dkk1 had no effect on Wnt5a-mediated neurite length of TH^+^ cells in E13.5 rat VM cultures (Fig. 5A,C,D), D4476 significantly inhibited the effects of Wnt5a on neurite length (Fig. 5A,C,E). Importantly, D4476 and Dkk1 had no effect on neurite length of TUJ^+^ neurons (data not shown), verifying the lack of toxicity of the antagonists. These results suggested that the action of Wnt5a on DA neurite length was mediated by non-canonical Wnt signaling.

We next examined whether blocking of the small GTPase, Rac1, a downstream component of the Wnt/PCP pathway in DA neurons [23] affected the total length of DA neurites in primary VM cultures. Treatment of control cultures with the Rac1 inhibitor, NSC23376 reduced TH+ neurite length (39%+4% reduction), presumably due to antagonism of endogenous Wnt signaling as well as other potential axon guidance pathways for which Rac1 is a downstream component. However, co-treatment with Wnt5a and NSC23766, significantly reduced total TH^+^ neurite length even further (60%±4%; Fig. 5A,C,F) compared to Wnt5a treatment alone. These findings suggest that at least in part Rac1, downstream of Wnt5a and thereby the PCP pathway, regulates DA neurite length. Finally, treatment with Wnt5a + D4476, but not Wnt5a + NSC23766, increased the number of TH^+^ neurites per cell (Fig. 5B), suggesting that while PCP signaling increases neurite length, other non-canonical Wnt signaling such as the Ca^2+^ pathway may cooperate to reduce the number of neurites.

### Wnt5a repels DA neurites in E11.5 and E14.5 explant cultures

**Figure 6:**
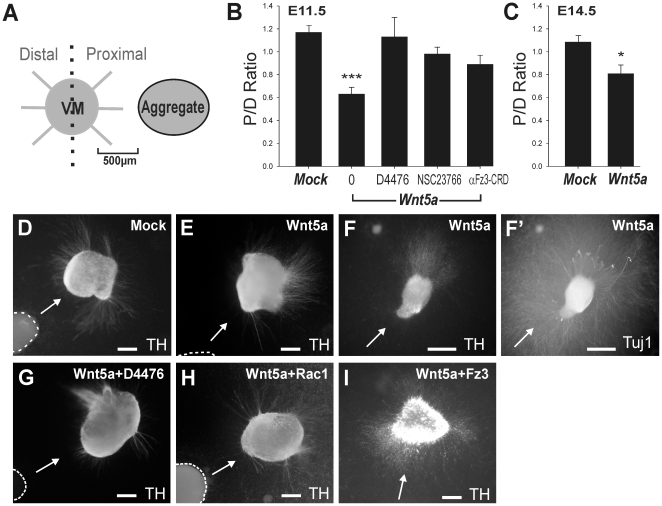
Wnt5a acts as a chemorepellant for DA neurites in VM explants. (**A**) Schematic representation of the explant cultures. VM explants were plated in collagen adjacent to mock- or Wnt5a-transfected cell aggregates. After three days in culture, the number of DA neurites (TH^+^) radiating from the proximal and distal sides of the VM explant (relative to the aggregate) were counted and expressed as a ratio. (**B**) In co-cultures of E11.5 mouse VM explants with Wnt5a-transfected cells, the majority of TH^+^ neurites emanated from the distal side of the explant, an effect that could be ablated by bath application of casein kinase 1 inhibitor D4476, Rac1 inhibitor NSC23766 and anti-Fz3-CRD. (**C**) The ability of Wnt5a to induce repulsion of DA neurites was maintained in older cultures (E14.5 mouse explants). Photomicrographs of E11.5 VM explants co-cultured with (**D**) mock-transfected cell aggregates and (**E**) Wnt5a-transfected cell aggregates. (**F**) VM explant co-cultured with Wnt5a cell aggregate, illustrating the specificity of Wnt5a to repel TH^+^ fibers (**F**) but not total neurite fibers (**F**', TUJ1-labeled fibers). (**G**–**I**) Images illustrating the effects of Wnt5a on neurite chemotaxis could be ablated by (**G**) D4476, (**H**) NSC23766 and (**I**) anti-Fz3-CRD. Dashed line demarcates the border of the cell aggregate. Arrow indicates direction of ligand signal (i.e. cell aggregate) relative to explant. Scale bar  = 200 µm.

After characterizing the effects of Wnt5a on neuritogenesis, we examined the chemoattractant or chemorepellant effect of Wnt5a on developing DA neurites in VM explants. In other CNS regions, Wnt5a repels neurites during development [31], [33], [35], but its effect on DA neurons has not yet been examined. Mouse VM explants (E11.5) were co-cultured with either mock-transfected or Wnt5a over-expressing cell aggregates for 72 hours and the number of TH^+^ fibers in the distal (D) and proximal (P) quadrants of the explant, with respect to the cell aggregate, were counted, as depicted in Fig. 6A. TH^+^ neurites from explants co-cultured with mock-transfected cell aggregates radiated out in all directions from the explant (Fig. 6D), showing a proximal to distal ratio of neurites close to 1 (1.19±0.06, n = 30; Fig. 6B). However, most TH^+^ neurites in VM explants cultured with Wnt5a-overexpressing cell aggregates emanated from the distal aspect of the explant, with a proximal to distal ratio of 0.70±0.09 (n = 29, p<0.001; Fig. 6B,E), indicating a repulsive effect. We confirmed that the effects of Wnt5a were specific to TH^+^ neurites by staining all neurites in culture (TUJ1^+^). Whilst TH^+^ fibers were repelled by Wnt5a (Fig. 6F), TUJ1^+^ neurites were observed emanating from all aspects of the same explants (Fig. 6F'). This effect of Wnt5a on DA neurites was ablated by bath application of the casein kinase 1 inhibitor D4476, the Rac1 inhibitor NSC23766, and anti-Fz3-CRD, indicating that the repulsive effects of Wnt5a on TH^+^ neurites are mediated by PCP/Wnt signaling via the Fz3 receptor (Fig. 6B,G–I). These findings have recently been supported by Fenstermaker et al (2010), who illustrated that DA neurons in Fz3(−/−) and Celsr3 (−/−) mice (Celsr3 being an additional component of the PCP pathway) were non-responsive to Wnt5a [55]. Given the contrasting effect of Wnt5a on neurite extension at differing developmental ages (E11.5 and E14.5, Fig. 2 and Fig. 3, respectively), we asked whether the chemorepulsion effect of Wnt5a on DA neurites was maintained in older VM explants (E14.5) and found that this was the case (Fig. 6C). We next investigated whether Wnt5a also regulates the development of midbrain DA axons in vivo and therefore examined the *Wnt5a* ^−/−^ mouse.

### Deletion of *Wnt5a* increases the number of TH^+^ fibers in the medial forebrain bundle and the innervation of the striatum in vivo

**Figure 7:**
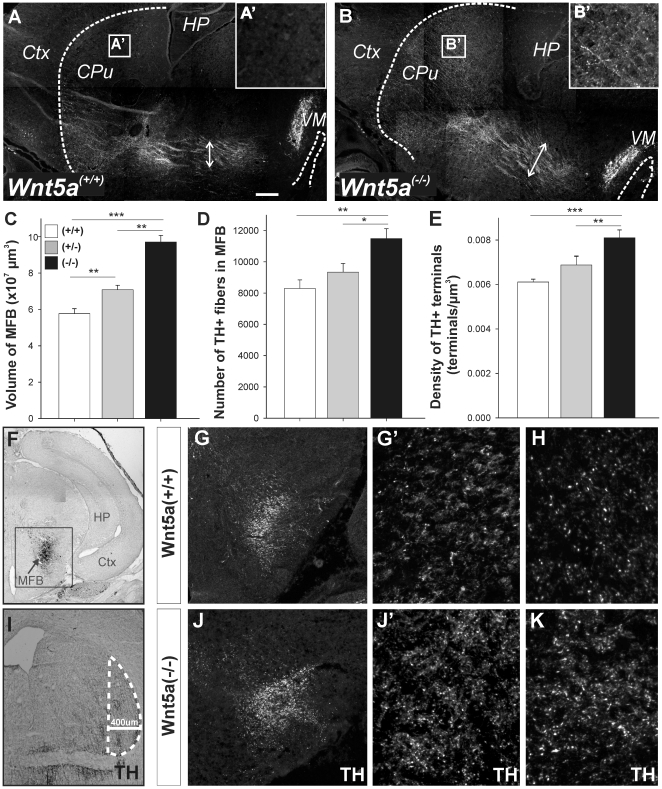
*Wnt5a* ^**−/−**^ mice display abnormal fasciculation of the DA axons in the median forebrain bundle (MFB) and changes in DA fiber and terminal density. (**A**) Sagittal image of the *Wnt5a* ^+/+^ and (**B**) *Wnt5a* ^−/−^ brain demonstrating morphological changes within the dopaminergic pathway. Note the broadening of the MFB in *Wnt5a* ^−/−^ compared to *Wnt5a* ^+/+^ mice, indicated by arrows, as well as increased terminal innervation in the dorsal striatum (**A**' and **B**'). (**C**) *Wnt5a* ^−/−^ mice showed defasciculation of the MFB as revealed by the increased volume occupied by TH^+^ fibers. (**D**) Within the MFB, *Wnt5a* ^−/−^ mice had significantly more TH^+^ fibers at proximal (320 µm) levels of the MFB. (**E**) *Wnt5a* ^−/−^ mice also had significantly more TH^+^ terminals within the lateral striatum than *Wnt5a^+/+^* mice. (**F**) Image illustrating the level of the MFB at which neurite counts were performed (HP, hippocampus; Ctx, cortex). (**I**) Image illustrating the area of the lateral striatum delineated for stereological estimates of TH^+^ terminal density. (**G**–**K**) Confocal photomicrographs illustrating differences in the midbrain dopaminergic pathway of *Wnt5a* ^+/+^ and *Wnt5a* ^−/−^ mice, respectively, including (**G,J**) broadening of the MFB, (**G**'**,J**') increased neurites within the MFB (320 µm from the DA neurons in the VM) and (**H,K**) increased terminal density in the lateral striatum. Insert in (**H**) illustrates individual TH^+^ terminals). Data represents mean ± s.e.m., n = 4–7 embryos/geneotype; * p<0.05, ** p<0.01, *** p<0.001.

The contribution of Wnt5a to DA axon growth and guidance was further examined by inspecting the trajectory of TH^+^ axons in the *Wnt5a* ^−/−^ mouse at E18. Gross examination of the pathway highlighted a broadening of the MFB and elaborated innervation in the dorsal striatum of *Wnt5a* ^−/−^ mice, not seen in *Wnt5a* ^+/+^ mice (Fig. 7A,B). Stereological quantification of the fiber bundle volume revealed that the *Wnt5a* ^−/−^ mice possessed a significantly enlarged MFB (9.708×10^7^ µm^3^±0.362×10^7^ µm^3^) compared to heterozygotes (7.081×10^7^ µm^3^±0.249×10^7^ µm^3^) and wildtype (5.770×10^7^ µm^3^±0.272×10^7^ µm^3^) littermates (Fig. 7C). Furthermore, there were more fibers within the caudal MFB (320 µm rostral to the TH^+^ neurons in the VM) of *Wnt5a* ^−/−^ mice than in *Wnt5a* ^+/+^ mice (11,487±632 and 8296±549 TH^+^ fibers, respectively; Fig. 7D,G,G',J,J'). The increase in TH^+^ fiber number was maintained within the MFB, with more rostral aspects of the bundle (800 µm rostral to the TH^+^ cells in the VM) also showing significant increases in fibers (data not shown).

We next used the density of TH+ synaptic varicosities in the striatum as a measure of the capacity of DA fibers to innervate their target structures. The lateral striatum of mutant and wildtype E18 embryos, which is innervated by both substantia nigra pars compacta (SNpc) and ventral tegmental area (VTA) DA neurons [62], [63] was examined. The terminal density in the lateral striatum of *Wnt5a* ^−/−^ mice (8.10×10^−3^±0.35×10^−3^ terminals/µm^3^) was significantly greater than in *Wnt5a* ^+/+^ mice (6.11×10^−3^±0.13×10^−3^ terminals/µm^3^, Fig. 7E,H,K) and innervation of the dorsal striatum in *Wnt5a* ^−/−^ mice (Fig. 7B,B') was notably denser than in wildtype littermates (Fig. 7A,A'). These results suggest that Wnt5a is required for the correct distribution of TH^+^ fibers in the dorso-lateral striatum, the target area of substantia nigra DA neurons.

Finally, in light of observed DA axon defects in E18 Wnt5a^−/−^ embryos, and our expression gradients identified in Figure 1, we examined an earlier time point (E12) to determine the effect of Wnt5a ablation on the establishment of the DA pathways. We observed that axons were notably shorter in Wnt5a^−/−^embryos (Figure S3A' and 3B', arrow heads), confirming the importance of Wnt5a in initial neurite elongation. Furthermore, broadening of the axon bundle was observed with fibers present in the intermediate zone and encroaching on the ventricular zone of the VM (Figure S3A” and 3B”), similar to E18 and validating Wnt5a's role in axon fasciculation.

## Discussion

Understanding the intricate and precise pattern of connectivity achieved during brain development has been, and remains, an outstanding challenge. This is particularly true for the dopaminergic pathways that arise from the ventral midbrain and innervate distant targets such as the striatum and cortex. Changes in the connectivity of these pathways and in the availability of their neurotransmitter, dopamine, underpin a number of neurological disorders including Parkinson's disease, schizophrenia and addiction. In this context, our study demonstrates a novel function for Wnt5a in the establishment of the dopaminergic pathways originating in the VM. First, we found that the temporal and spatial expression pattern of Wnt5a correlates with the development of the nigrostriatal/mesolimbic DA pathways. Second, we report that Wnt5a selectively regulates the axon length of TH^+^ cells in primary VM cultures in a time-dependent manner, promoting axon extension at E11.5 (and rat E13.5) and axon retraction at E14.5. Third, we identify Wnt5a as a chemorepellant of DA neurites in mouse E11.5 and E14.5 explant cultures. We confirm that these growth and chemotaxic effects of Wnt5a are mediated by Frizzled and involve the activation of Rac1, a component of the Wnt/PCP pathway. Fourth, analysis of the *Wnt5a* ^−/−^ mice revealed that Wnt5a is required for fasciculation of DA axons in the MFB, and for the innervation of the dorsolateral striatum, the target area of substantia nigra DA neurons.

**Figure 8:**
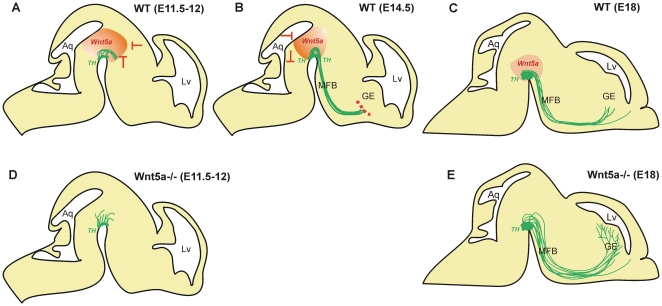
Model of Wnt5a's role in the development of the DA mesostriatal/mesolimbic pathways in mice. (**A**) In wildtype mice, at E11.5-12, a high ventricular to low mantle zone expression of Wnt5a (red shading) in the ventral midbrain, combined with our in vitro findings of Wnt5a's ability to elongate and repel DA axons (green), suggests that Wnt5a may be capable of polarizing and driving DA axons out of the VM. Higher Wnt5a levels in the rostral VM (red), in combination with its chemorepulsive function, further suggests that Wnt5a may prevent premature rostral trajectory of these DA axons. (**B**) At E14.5, a higher caudal to lower rostral Wnt5a gradient (red), combined with the maintained repulsive action of Wnt5a at this age, may ensure TH^+^ axons maintain their forebrain trajectory. The ability of Wnt5a to induce axon retraction in vitro (as shown in Fig. 3) suggests a plausible role in axon stalling, a key developmental event that prevents axons prematurely entering their forebrain striatal targets. The dotted red line indicates the border of the ganglionic eminence where axon stalling occurs. (**C**) At E18, low expression of Wnt5a is maintained within the VM. At this stage in development TH^+^ axons are present within the GE and are making increasing synaptic contacts. (**D**) In the absence of Wnt5a, at E11.5-12, TH^+^ axons maintain a rostral projection but are shorter and show a lack of organization. (**E**) By E18, the disorganization of TH^+^ fibers is evident by the defasiculation of the MFB in Wnt5a^−/−^ mice. Additionally, axons prematurely enter the target, presumably due to loss of axonal stalling, resulting in increased striatal innervation in Wnt5a^−/−^ mice. Lv, lateral ventricle; Aq, aquaduct; GE, ganglionic eminence; MFB, medial forebrain bundle.

Wnt5a expression within the VM and caudal region of the MFB was maintained during development of the DA pathways. More precise temporal and spatial gradients reflect the functions of this protein in DA axon morphogenesis. We speculate that at E11.5, the high expression of Wnt5a within the ventricular zone and in the rostral part of the ventral midbrain, (Fig. 1A,E) may prevent axons from entering the ventricular zone and from taking a premature anterior direction. In support, our in vitro results at E11.5 indicate that Wnt5a increases neurite length during initial DA axon development (Fig. 2D,E), and repels them away from the source of Wnt5a (Fig. 6B,D,E). Additionally, E12 Wnt5a^−/−^embryos show reduced neurite length and disorganization of DA neurites, with fibers seen within intermediate layers of the VM (Figure S3). We therefore suggest that the initial effect of Wnt5a is to contribute to DA axonal elongation and to maintain axons within the VM, but out of the ventricular zone (Fig. 8A, D). Later in development, by E14.5, the high rostral expression of Wnt5a is down-regulated and expression is higher in the caudal VM (Fig. 1L). This gradient shift may facilitate DA axons taking a forebrain trajectory by being repelled away from the caudal VM, and preventing their entry into the hindbrain (Fig. 8B). Interestingly, whilst not observed by us, Fenstermaker et al (2010) reported the appearance of DA axons in the hindbrain of Wnt5a^−/−^mice at E12.5, a phenotype that was lost later in development [55], yet supports our theory of Wnt5a ensuring DA axons maintain their rostral trajectory.

Previous studies have shown that Fz3 is involved in PCP signaling in the context of axon guidance [64] and that Fz3-deficient mice, whilst possessing normal numbers of DA neurons, have no nigrostriatal pathway [57], [58]. Moreover, Stuebner et al. has recently reported that Fz3 and Fz6 cooperate to regulate midbrain morphogenesis [65]. Similarly, we have previously reported that Wnt5a mutant mice have a near normal number of DA neurons, but show a clear defect in midbrain morphogenesis [23]. We hereby report that, similar to Fz3, Wnt5a promotes DA neuritogenesis *in vitro* and that *Wnt5a* ^−/−^ mice exhibit defects in DA axonogenesis. Moreover, we found that αFz3-CRD blocked the neuritogenic and repulsive effects of Wnt5a, suggesting that these Wnt5a effects are mediated by Fz3 or Fz3-like receptors. Fenstermaker et al., (2010) has since illustrated notable defects within the trajectory of DA fibers in Fz3^−/−^mice, observing several fibers projecting caudally and failing to make striatal contacts [55]. Surprisingly, we observed increased fiber innervation in Wnt5a^−/−^ mice suggesting that Fz3 is only partially responsible for our observations. In the future, analysis of double mutant mice for Fz3 and Wnt5a may more clearly identify these roles and highlight the need to investigate other Wnt related receptors.

Signaling mediated through Frizzled receptors is commonly associated with axon elongation and attraction while, in contrast, signaling through Ryk induces axon repulsion [25]–[30], [66]. However, Ryk receptors activated by Wnt5a gradients promoted elongation of cortical axons in the callosal and corticospinal tract [31], [33], [35], and chemorepelled these same axons via Wnt5a signaling through both Ryk and Frizzled receptors. These differing actions were mediated by Ca^2+^ changes involving IP3 receptors and/or TRP channels [33]. Whilst we can not directly attribute the effects of Wnt5a to Ryk binding, the high expression of Ryk within DA neurons of the VM, combined with RYK-Fc antagonism in a DA cell line and existing literature in other pathways, suggests that Ryk may also play a role in regulating DA morphogenesis. Analysis of Ryk-deficient mice as well as double mutants (Wnt5a and Ryk) or siRNA experiments may shed more light on this receptor's involvement in these processes.

Whilst a number of guidance molecules have been recognized for their role in DA neurite development, few studies have verified these functions in vivo. In our study, we examined the nigrostriatal DA pathway in *Wnt5a* ^−/−^ mice, validating a number of our in vitro findings and the importance of Wnt5a in DA connectivity. In wildtype mice, TH^+^ axons remain tightly fasciculated within the MFB as they project towards their forebrain targets. In contrast, DA axons in the *Wnt5a* ^−/−^ mice were clearly defasciculated throughout the MFB, with broadening of the bundle observed from caudal to rostral levels of the pathway. Interestingly, defasciculation and broadening of the MFB has also been observed in *Sema3F* ^−/−^ mice [9] and *Ryk* ^−/−^ mice showed defasciculation of the callosal axon bundle crossing the midline [35]. These findings indicate that Wnt5a is required to fasciculate DA axons in vivo.

Surprisingly, despite the role of Wnt5a in promoting axonal growth, repelling DA axons and regulating fasciculation of the DA fiber bundle, DA axons in *Wnt5a* ^−/−^ mice maintained their appropriate caudo-rostral trajectory, indicating that Wnt5a alone is not necessary to initiate neurite growth or provide directionality in vivo. Collectively these findings suggest that other guidance molecules or Wnts might be capable of compensating for the lack of Wnt5a during the establishment of this pathway. However, more axons were detected within the MFB of *Wnt5a* ^−/−^ mice. Since we previously reported that the number of neurons in *Wnt5a* ^−/−^ and *Wnt5a* ^+/+^ mice is not different [23], the increased fiber density in the MFB of *Wnt5a* ^−/−^ mice suggests a possible increase in DA neurites per DA neuron, or alternatively, increased branching of the DA axons. Interestingly, our in vitro data supports both possibilities, but the greater effect on branching suggests a predominant role of Wnt5a on DA neurite branching, rather than on the number of neurites (Fig. 2F,G).

Finally, it is important to note that despite DA axons arriving at the border of the ganglionic eminence at approximately E15 in rats, they do not enter the striatum, but rather increase in numbers until approximately E17 (comparable to mouse E15) [1]. In support of a possible role for Wnt5a in axon stalling, we found that Wnt5a had a negative effect on DA neurite length in vitro, at E14.5 (approximately equivalent to rat E16.5) (Fig. 3), and that axonal innervation of the dorsolateral striatum was accelerated in the *Wnt5a* ^−/−^ mice. These results indicate that Wnt5a may be the repulsive signal that prevents premature entry of DA axons into the striatum. Indeed, both the increased fiber number in the MFB and the dense striatal innervation of the *Wnt5a* ^−/−^ mice may reflect a premature maturation of the pathway (i.e. loss of axonal stalling). Thus our results suggest that one of the functions of Wnt5a in the nigrostriatal system would be to prevent the premature maturation of this pathway.

In summary, our findings identify a number of key roles for Wnt5a in the development of DA axons in vitro and in the maturation of the MFB in vivo. Wnt5a promotes DA axon elongation, retraction and repulsion in a time-dependent manner, as well as maturation and fasciculation of the MFB. These effects, at least in part, are mediated through the Frizzled receptors and downstream activation of the Wnt/PCP pathway. Whilst broadening our knowledge of dopamine development, an understanding of the regulation and promotion of DA axonal growth and guidance may have significant implications for a number of neurological disorders in which the development of nigrostriatal DA axons are affected, as well as enhancing integration of grafted dopamine neurons into the Parkinsonian brain.

## Supporting Information

Figure S1 **Schematic representation of the site of action of the antagonists employed to identify the pathways mediating the effects of Wnt5a.** sFRP, αWnt5a and RYK-Fc act to sequester Wnt5a out of circulation, thereby preventing its interaction with Wnt-related receptors. αFz3-CRD binds directly to the frizzled-3 receptor, thus preventing Wnt-receptor interaction. Dkk1 does not bind Wnt but affects the interaction of Wnt with the LRP co-receptor, thereby affecting canonical Wnt signaling. D4476, a casein kinase 1 antagonist, blocks the Wnt activity-dependent phosphorylation of Dishevelled and thereby prevents downstream canonical and non-canonical Wnt signaling. NSC23766 is a Rac1 antagonist and thereby an inhibitor of the non-canonical Wnt/PCP pathway. CK1, casein kinase 1; Dkk1, Dickkopf-1; Fz, Frizzled; LRP5/6, low density lipoprotein receptor-related protein 5/6; αFz3-CRD, Fz3 antibody; αWnt5a, Wnt5a antibody.
(TIF)

Figure S2 **Effects of Wnt receptor antagonism on neurites of all VM neurons.** Immunocytochemistry for all neurons (TUJ1^+^) revealed that (**A**) αFz3-CRD and (**B**) RYK-Fc had no effect on neurite length of non-TH^+^ neurons within the VM, indicating that the effects seen in Fig. 5 were specific to DA neurites. Furthermore, the absence of an effect of αFz3-CRD and RYK-Fc on the general neuronal population verifies the lack of toxicity of these proteins at the doses selected.
(TIF)

Figure S3 **Young** ***Wnt5a*** **^−/−^ embryos display abnormal DA axon length and fasciculation.** Sagittal images illustrating TH^+^ axons in the VM of (**A**) wildtype and (**B**) Wnt5a knockout littermates. (A', A” and B', B”) represent higher magnification of the VM depicted in (**A**) and (**B**). Images show that compared to *Wnt5a ^+/+^* mice, TH^+^ axons in *Wnt5a ^−/−^* mice are shorter (A' and B', arrow heads) and less organized/tightly fasciculated (A”, B”, arrows).
(TIF)

## References

[1] Van den Heuvel DM, Pasterkamp RJ (2008). Getting connected in the dopamine system.. Prog Neurobiol.

[2] Sieber BA, Kuzmin A, Canals JM, Danielsson A, Paratcha G (2004). Disruption of EphA/ephrin-a signaling in the nigrostriatal system
reduces dopaminergic innervation and dissociates behavioral responses to
amphetamine and cocaine.. Mol Cell Neurosci.

[3] Yue Y, Widmer DA, Halladay AK, Cerretti DP, Wagner GC (1999). Specification of distinct dopaminergic neural pathways: roles of
the Eph family receptor EphB1 and ligand ephrin-B2.. J Neurosci.

[4] Richards AB, Scheel TA, Wang K, Henkemeyer M, Kromer LF (2007). EphB1 null mice exhibit neuronal loss in substantia nigra pars
reticulata and spontaneous locomotor hyperactivity.. Eur J Neurosci.

[5] Kawano H, Horie M, Honma S, Kawamura K, Takeuchi K (2003). Aberrant trajectory of ascending dopaminergic pathway in mice
lacking Nkx2.1.. Exp Neurol.

[6] Arroyave Hernandez CM, Echevarria Pinto M, Hernandez Montiel HL (2007). Food allergy mediated by IgG antibodies associated with migraine
in adults.. Rev Alerg Mex.

[7] Garcia C, Aranda J, Arnold E, Thebault S, Macotela Y (2008). Vasoinhibins prevent retinal vasopermeability associated with
diabetic retinopathy in rats via protein phosphatase 2A-dependent eNOS
inactivation.. J Clin Invest.

[8] Hernandez-Montiel HL, Tamariz E, Sandoval-Minero MT, Varela-Echavarria A (2008). Semaphorins 3A, 3C, and 3F in mesencephalic dopaminergic axon
pathfinding.. J Comp Neurol.

[9] Kolk SM, Gunput RA, Tran TS, van den Heuvel DM, Prasad AA (2009). Semaphorin 3F is a bifunctional guidance cue for dopaminergic
axons and controls their fasciculation, channeling, rostral growth, and
intracortical targeting.. J Neurosci.

[10] Lin L, Rao Y, Isacson O (2005). Netrin-1 and slit-2 regulate and direct neurite growth of ventral
midbrain dopaminergic neurons.. Mol Cell Neurosci.

[11] Lin L, Isacson O (2006). Axonal growth regulation of fetal and embryonic stem cell-derived
dopaminergic neurons by Netrin-1 and Slits.. Stem Cells.

[12] Saueressig H, Burrill J, Goulding M (1999). Engrailed-1 and netrin-1 regulate axon pathfinding by association
interneurons that project to motor neurons.. Development.

[13] Alberi L, Sgado P, Simon HH (2004). Engrailed genes are cell-autonomously required to prevent
apoptosis in mesencephalic dopaminergic neurons.. Development.

[14] Hammond R, Blaess S, Abeliovich A (2009). Sonic hedgehog is a chemoattractant for midbrain dopaminergic
axons.. PLoS One.

[15] McMahon AP, Bradley A (1990). The Wnt-1 (int-1) proto-oncogene is required for development of a
large region of the mouse brain.. Cell.

[16] Thomas KR, Capecchi MR (1990). Targeted disruption of the murine int-1 proto-oncogene resulting
in severe abnormalities in midbrain and cerebellar
development.. Nature.

[17] Danielian PS, McMahon AP (1996). Engrailed-1 as a target of the Wnt-1 signalling pathway in
vertebrate midbrain development.. Nature.

[18] Castelo-Branco G, Arenas E (2006). Function of Wnts in dopaminergic neuron
development.. Neurodegener Dis.

[19] Schulte G, Bryja V, Rawal N, Castelo-Branco G, Sousa KM (2005). Purified Wnt-5a increases differentiation of midbrain
dopaminergic cells and dishevelled phosphorylation.. J Neurochem.

[20] Parish CL, Castelo-Branco G, Rawal N, Tonnesen J, Sorensen AT (2008). Wnt5a-treated midbrain neural stem cells improve dopamine cell
replacement therapy in parkinsonian mice.. J Clin Invest.

[21] Castelo-Branco G, Wagner J, Rodriguez FJ, Kele J, Sousa K (2003). Differential regulation of midbrain dopaminergic neuron
development by Wnt-1, Wnt-3a, and Wnt-5a.. Proc Natl Acad Sci U S A.

[22] Castelo-Branco G, Sousa KM, Bryja V, Pinto L, Wagner J (2006). Ventral midbrain glia express region-specific transcription
factors and regulate dopaminergic neurogenesis through Wnt-5a
secretion.. Mol Cell Neurosci.

[23] Andersson ER, Prakash N, Cajanek L, Minina E, Bryja V (2008). Wnt5a regulates ventral midbrain morphogenesis and the
development of A9-A10 dopaminergic cells in vivo.. PLoS ONE.

[24] Prakash N, Brodski C, Naserke T, Puelles E, Gogoi R (2006). A Wnt1-regulated genetic network controls the identity and fate
of midbrain-dopaminergic progenitors in vivo.. Development.

[25] Zou Y (2004). Wnt signaling in axon guidance.. Trends Neurosci.

[26] Endo Y, Rubin JS (2007). Wnt signaling and neurite outgrowth: insights and
questions.. Cancer Sci.

[27] Bovolenta P, Rodriguez J, Esteve P (2006). Frizzled/RYK mediated signalling in axon
guidance.. Development.

[28] Ille F, Sommer L (2005). Wnt signaling: multiple functions in neural
development.. Cell Mol Life Sci.

[29] Lu W, Yamamoto V, Ortega B, Baltimore D (2004). Mammalian Ryk is a Wnt coreceptor required for stimulation of
neurite outgrowth.. Cell.

[30] Speese SD, Budnik V (2007). Wnts: up-and-coming at the synapse.. Trends Neurosci.

[31] Liu Y, Shi J, Lu CC, Wang ZB, Lyuksyutova AI (2005). Ryk-mediated Wnt repulsion regulates posterior-directed growth of
corticospinal tract.. Nat Neurosci.

[32] Zou Y, Lyuksyutova AI (2007). Morphogens as conserved axon guidance cues.. Curr Opin Neurobiol.

[33] Li L, Hutchins BI, Kalil K (2009). Wnt5a induces simultaneous cortical axon outgrowth and repulsive
axon guidance through distinct signaling mechanisms.. J Neurosci.

[34] Yoshikawa S, McKinnon RD, Kokel M, Thomas JB (2003). Wnt-mediated axon guidance via the Drosophila Derailed
receptor.. Nature.

[35] Keeble TR, Halford MM, Seaman C, Kee N, Macheda M (2006). The Wnt receptor Ryk is required for Wnt5a-mediated axon guidance
on the contralateral side of the corpus callosum.. J Neurosci.

[36] Keeble TR, Cooper HM (2006). Ryk: a novel Wnt receptor regulating axon
pathfinding.. Int J Biochem Cell Biol.

[37] Mikels AJ, Nusse R (2006). Purified Wnt5a protein activates or inhibits beta-catenin-TCF
signaling depending on receptor context.. PLoS Biol.

[38] Qian D, Jones C, Rzadzinska A, Mark S, Zhang X (2007). Wnt5a functions in planar cell polarity regulation in
mice.. Dev Biol.

[39] Witze ES, Litman ES, Argast GM, Moon RT, Ahn NG (2008). Wnt5a control of cell polarity and directional movement by
polarized redistribution of adhesion receptors.. Science.

[40] Yamaguchi TP, Bradley A, McMahon AP, Jones S (1999). A Wnt5a pathway underlies outgrowth of multiple structures in the
vertebrate embryo.. Development.

[41] Kele J, Simplicio N, Ferri AL, Mira H, Guillemot F (2006). Neurogenin 2 is required for the development of ventral midbrain
dopaminergic neurons.. Development.

[42] Sawamoto K, Nakao N, Kobayashi K, Matsushita N, Takahashi H (2001). Visualization, direct isolation, and transplantation of midbrain
dopaminergic neurons.. Proc Natl Acad Sci U S A.

[43] Rawal N, Castelo-Branco G, Sousa KM, Kele J, Kobayashi K (2006). Dynamic temporal and cell type-specific expression of Wnt
signaling components in the developing midbrain.. Exp Cell Res.

[44] Pfaffl MW (2001). A new mathematical model for relative quantification in real-time
RT-PCR.. Nucleic Acids Res.

[45] Stacker SA, Stenvers K, Caesar C, Vitali A, Domagala T (1999). Biosynthesis of vascular endothelial growth factor-D involves
proteolytic processing which generates non-covalent
homodimers.. J Biol Chem.

[46] Fuentes EO, Leemhuis J, Stark GB, Lang EM (2008). Rho kinase inhibitors Y27632 and H1152 augment neurite extension
in the presence of cultured Schwann cells.. J Brachial Plex Peripher Nerve Inj.

[47] Turner BJ, Parkinson NJ, Davies KE, Talbot K (2009). Survival motor neuron deficiency enhances progression in an
amyotrophic lateral sclerosis mouse model.. Neurobiol Dis.

[48] Snyder EY, Deitcher DL, Walsh C, Arnold-Aldea S, Hartwieg EA (1992). Multipotent neural cell lines can engraft and participate in
development of mouse cerebellum.. Cell.

[49] Gundersen HJ, Bagger P, Bendtsen TF, Evans SM, Korbo L (1988). The new stereological tools: disector, fractionator, nucleator
and point sampled intercepts and their use in pathological research and
diagnosis.. APMIS.

[50] West MJ, Slomianka L, Gundersen HJ (1991). Unbiased stereological estimation of the total number of neurons
in thesubdivisions of the rat hippocampus using the optical
fractionator.. Anatomical Record.

[51] Parish CL, Finkelstein DI, Drago J, Borrelli E, Horne MK (2001). The role of dopamine receptors in regulating the size of axonal
arbors.. J Neurosci.

[52] West MJ, Gundersen HJ (1990). Unbiased stereological estimation of the number of neurons in the
human hippocampus.. Journal of Comparative Neurology.

[53] Braendgaard H, Evans SM, Howard CV, Gundersen HJ (1990). The total number of neurons in the human neocortex unbiasedly
estimated using optical disectors.. Journal of Microscopy.

[54] Nakamura S, Ito Y, Shirasaki R, Murakami F (2000). Local directional cues control growth polarity of dopaminergic
axons along the rostrocaudal axis.. J Neurosci.

[55] Fenstermaker AG, Prasad AA, Bechara A, Adolfs Y, Tissir F (2010). Wnt/planar cell polarity signaling controls the
anterior-posterior organization of monoaminergic axons in the
brainstem.. J Neurosci.

[56] Wagner J, Akerud P, Castro DS, Holm PC, Canals JM (1999). Induction of a midbrain dopaminergic phenotype in
Nurr1-overexpressing neural stem cells by type 1 astrocytes.. Nat Biotechnol.

[57] Wang Y, Thekdi N, Smallwood PM, Macke JP, Nathans J (2002). Frizzled-3 is required for the development of major fiber tracts
in the rostral CNS.. J Neurosci.

[58] Wang Y, Zhang J, Mori S, Nathans J (2006). Axonal growth and guidance defects in Frizzled3 knock-out mice: a
comparison of diffusion tensor magnetic resonance imaging, neurofilament
staining, and genetically directed cell labeling.. J Neurosci.

[59] Bodmer D, Levine-Wilkinson S, Richmond A, Hirsh S, Kuruvilla R (2009). Wnt5a mediates nerve growth factor-dependent axonal branching and
growth in developing sympathetic neurons.. J Neurosci.

[60] Glinka A, Wu W, Delius H, Monaghan AP, Blumenstock C (1998). Dickkopf-1 is a member of a new family of secreted proteins and
functions in head induction.. Nature.

[61] Bryja V, Schulte G, Arenas E (2007). Wnt-3a utilizes a novel low dose and rapid pathway that does not
require casein kinase 1-mediated phosphorylation of Dvl to activate
beta-catenin.. Cell Signal.

[62] Fallon JH, Moore RY (1978). Catecholamine innervation of the basal forebrain. IV. Topography
of the dopamine projection to the basal forebrain and
neostriatum.. J Comp Neurol.

[63] Bjorklund A, Lindvall O, Bjorklund A, Hokfelt T (1984). Dopamine-containing systems in the CNS;.

[64] Wang Y, Nathans J (2007). Tissue/planar cell polarity in vertebrates: new insights and new
questions.. Development.

[65] Stuebner S, Faus-Kessler T, Fischer T, Wurst W, Prakash N (2009). Fzd3 and Fzd6 deficiency results in a severe midbrain
morphogenesis defect.. Dev Dyn.

[66] Sanchez-Camacho C, Bovolenta P (2009). Emerging mechanisms in morphogen-mediated axon
guidance.. Bioessays.

