# Supplementary figures and images for: Wnt5a Regulates Midbrain Dopaminergic Axon Growth and Guidance
Source: PLoS One. 2011 Mar 31;6(3):e18373. doi: 10.1371/journal.pone.0018373 (PMC3069098; doi:10.1371/journal.pone.0018373)

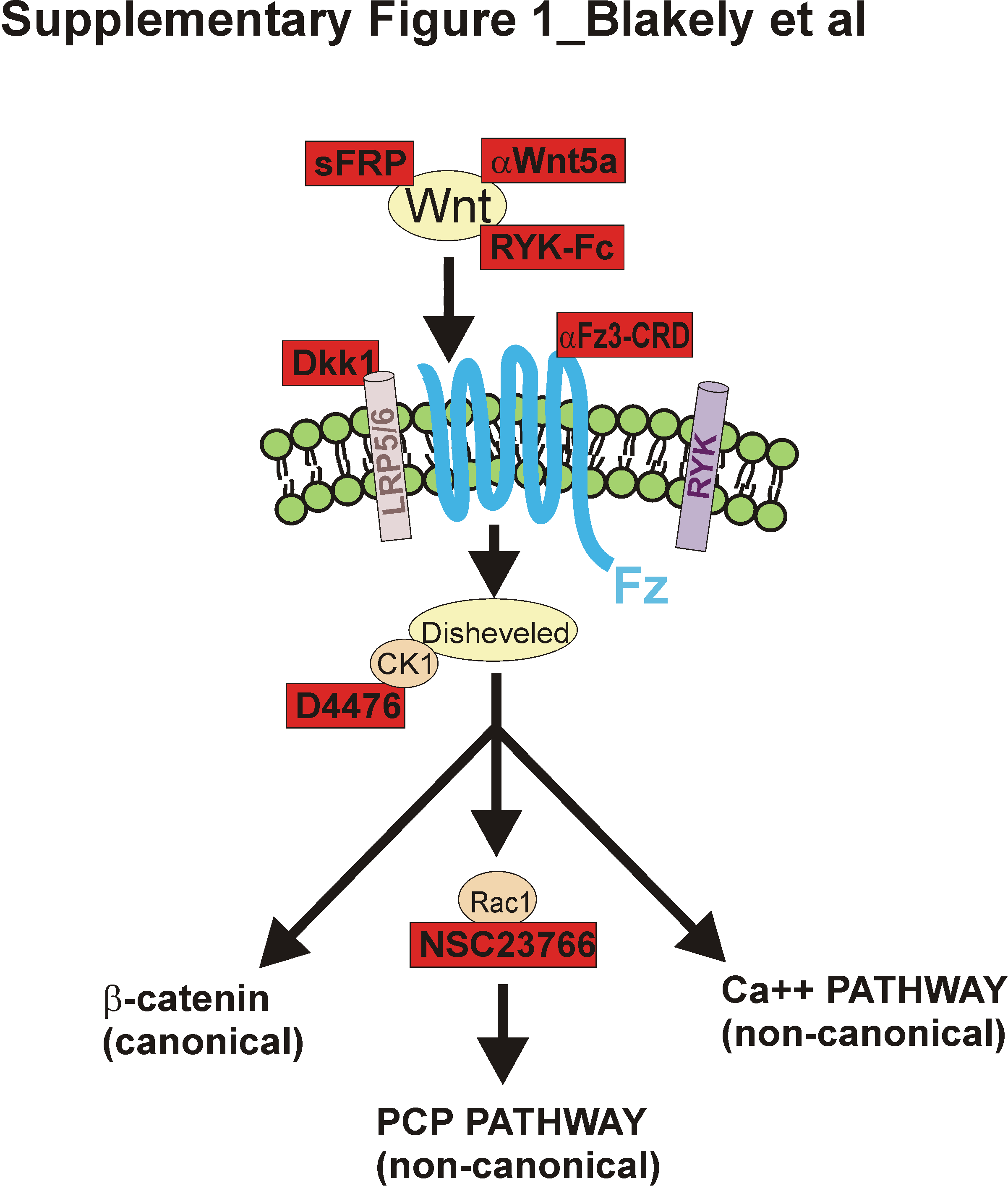

Supplement: Figure S1 — Schematic representation of the site of action of the antagonists employed to identify the pathways mediating the effects of Wnt5a. sFRP, αWnt5a and RYK-Fc act to sequester Wnt5a out of circulation, thereby preventing its interaction with Wnt-related receptors. αFz3-CRD binds directly to the frizzled-3 receptor, thus preventing Wnt-receptor interaction. Dkk1 does not bind Wnt but affects the interaction of Wnt with the LRP co-receptor, thereby affecting canonical Wnt signaling. D4476, a casein kinase 1 antagonist, blocks the Wnt activity-dependent phosphorylation of Dishevelled and thereby prevents downstream canonical and non-canonical Wnt signaling. NSC23766 is a Rac1 antagonist and thereby an inhibitor of the non-canonical Wnt/PCP pathway. CK1, casein kinase 1; Dkk1, Dickkopf-1; Fz, Frizzled; LRP5/6, low density lipoprotein receptor-related protein 5/6; αFz3-CRD, Fz3 antibody; αWnt5a, Wnt5a antibody. (TIF) [file pone.0018373.s001.tif]

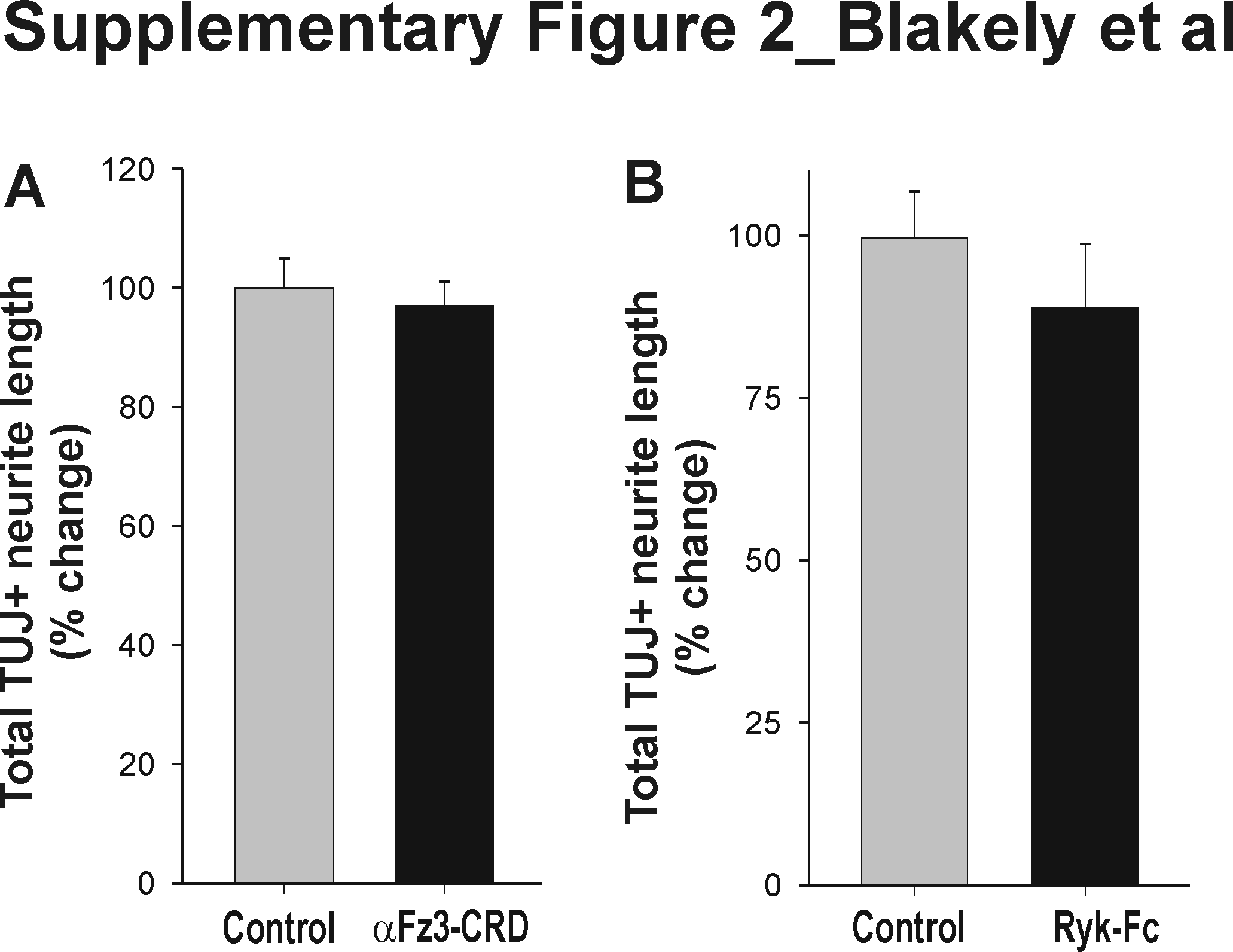

Supplement: Figure S2 — Effects of Wnt receptor antagonism on neurites of all VM neurons. Immunocytochemistry for all neurons (TUJ1+) revealed that (A) αFz3-CRD and (B) RYK-Fc had no effect on neurite length of non-TH+ neurons within the VM, indicating that the effects seen in Fig. 5 were specific to DA neurites. Furthermore, the absence of an effect of αFz3-CRD and RYK-Fc on the general neuronal population verifies the lack of toxicity of these proteins at the doses selected. (TIF) [file pone.0018373.s002.tif]

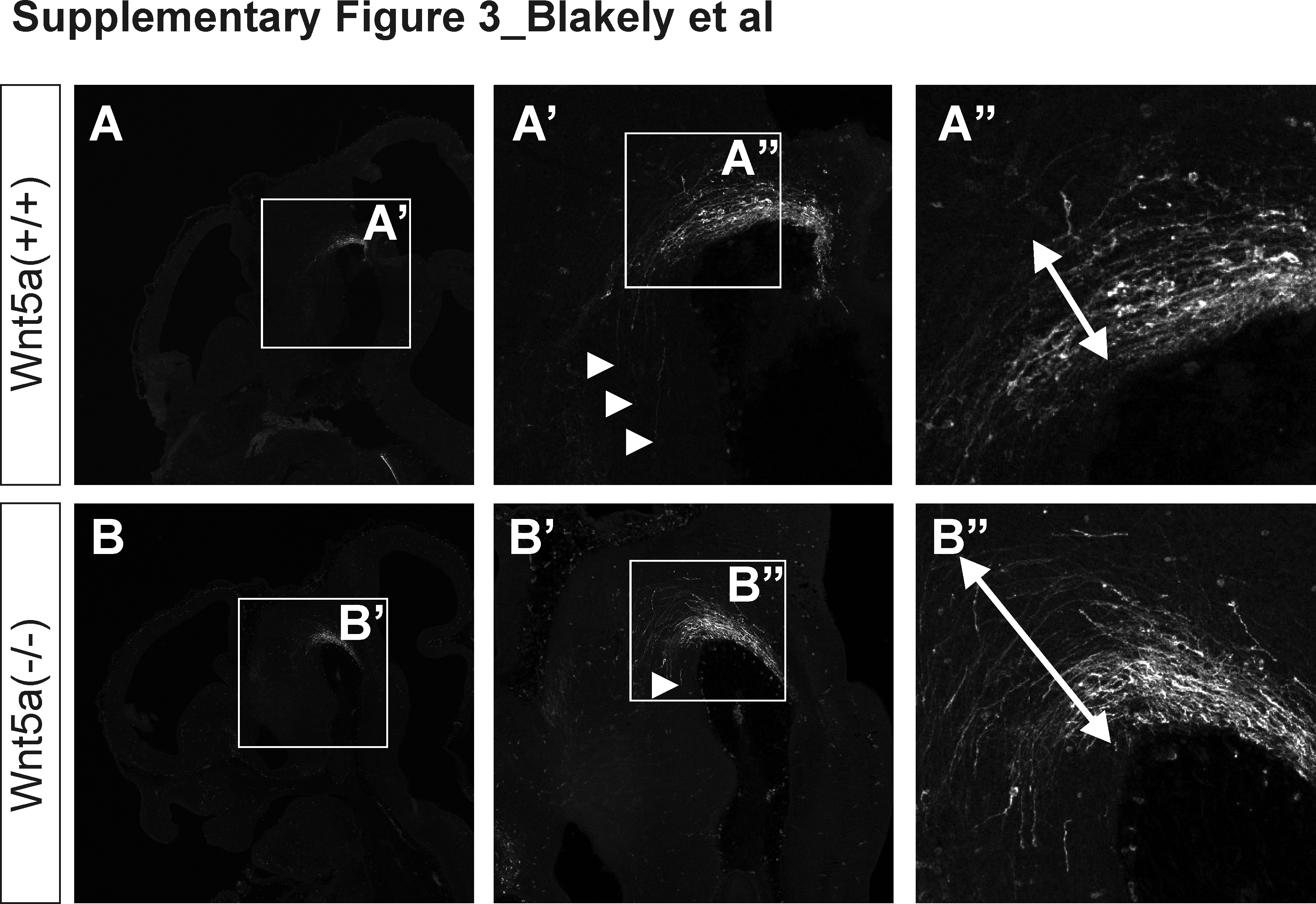

Supplement: Figure S3 — Young Wnt5a −/− embryos display abnormal DA axon length and fasciculation. Sagittal images illustrating TH+ axons in the VM of (A) wildtype and (B) Wnt5a knockout littermates. (A', A” and B', B”) represent higher magnification of the VM depicted in (A) and (B). Images show that compared to Wnt5a +/+ mice, TH+ axons in Wnt5a −/− mice are shorter (A' and B', arrow heads) and less organized/tightly fasciculated (A”, B”, arrows). (TIF) [file pone.0018373.s003.tif]
